# Proteomic and metabolomic characterizations of moyamoya disease patient sera

**DOI:** 10.1002/brb3.3328

**Published:** 2023-11-14

**Authors:** Qingbao Guo, Qian‐Nan Wang, Jingjie Li, Simeng Liu, Xiaopeng Wang, Dan Yu, Zheng‐Xing Zou, Gan Gao, Qian Zhang, Fang‐Bin Hao, Jie Feng, Ri‐Miao Yang, Minjie Wang, Heguan Fu, Xiangyang Bao, Lian Duan

**Affiliations:** ^1^ Medical School of Chinese PLA Beijing China; ^2^ Department of Neurosurgery, The First Medical Centre Chinese PLA General Hospital Beijing China; ^3^ Department of Neurosurgery, The Fifth Medical Centre Chinese PLA General Hospital Beijing China; ^4^ Department of Neurosurgery, The Eighth Medical Centre Chinese PLA General Hospital Beijing China

**Keywords:** integration analysis, metabolome, moyamoya disease, proteome

## Abstract

**Background:**

The pathogenesis of moyamoya disease (MMD) is unclear. Inflammation and immune imbalance have been identified as potential factors contributing to the occurrence and progression of MMD. However, the specific proteins and metabolites responsible for triggering this process are yet to be established. The purpose of this study is to identify differentially expressed proteins and metabolites in patients with MMD and perform Kyoto Encyclopedia of Genes and Genomes pathway integration analysis to pinpoint crucial proteins and metabolites involved in the disease.

**Methods:**

We performed untargeted metabolomic and data‐independent acquisition proteomic analyses on the serum samples of individuals with MMD and healthy controls (HC).

**Results:**

In patients with MMD versus HC, 24 proteins and 60 metabolites, including 21 anionic metabolites and 39 cationic metabolites, which were significantly different, were identified. In patients with MMD, several proteins involved in inflammation and immune metabolism, such as tubulin beta‐6 and complement C4, were found to have significantly altered levels. Similarly, many metabolites involved in inflammation and immune metabolisms, such as dimethyl 4‐hydroxyisophthalate, beta‐nicotinamide mononucleotide, 2‐(3‐(4‐pyridyl)‐1*H*‐1,2,4‐triazol‐5‐yl)pyridine, and PC (17:1/18:2), were significantly altered. Intriguingly, these proteins and metabolites are involved in the progression of atherosclerosis through immune and inflammatory pathways, although some have never been reported in MMD. Moreover, integrated proteomics and metabolomics studies were conducted to determine shared pathways involving cholesterol metabolism, vitamin digestion, fat digestion, and absorption pathways of proteins and metabolites, which warrant further investigation.

**Conclusions:**

Significant increases in pro‐inflammatory and immunosuppressive abilities have been observed in patients with MMD, accompanied by significant reductions in anti‐inflammatory and immune regulation. Various metabolites and proteins implicated in these processes have been identified for the first time. These findings hold immense significance for comprehending the pathogenesis of MMD and for the development of future drug therapies.

## Abstract 2

Significant increases in pro‐inflammatory and immunosuppressive abilities have been observed in patients with MMD, accompanied by significant reductions in anti‐inflammatory and immune regulation. Various metabolites and proteins implicated in these processes have been identified for the first time. These findings hold immense significance for comprehending the pathogenesis of moyamoya disease and for the development of future drug therapies.

## 1 INTRODUCTION

Moyamoya disease (MMD) is a rare and chronic occlusive cerebrovascular disease characterized by spontaneous, unilateral, or bilateral, and progressive steno‐occlusive changes in the distal internal carotid artery and its major branches, resulting in the formation of an abnormally compensatory collateral vascular network (Ihara et al., 2022). MMD was originally thought to mainly affect people of Asian heritage, but now it can be found in people with many ethnic backgrounds worldwide, including American and European populations (Caldarelli et al., 2001; Suzuki & Kodama, 1983). The incidence peaked in two age groups: children aged approximately 5 years and adults in their mid‐40s (Baba et al., 2008; Han et al., 2000). The female‐to‐male ratio was approximately 2:1 (Baba et al., 2008; Scott et al., 2004). In Japan, the prevalence rate of MMD in children is approximately 3 cases per 100,000 children, making it the most common cerebrovascular disease in children (Baba et al., 2008; Han et al., 2000; Nagaraja et al., 1994). The total incidence rate of MMD in patients in Europe is approximately one tenth of that in Japan (Yonekawa et al., 1997).

Since its first report in the 1950s, numerous studies have focused on the epidemiological and clinical characteristics of MMD in different regions. Given the contrast between poor response to pharmacotherapy and documented surgical success, surgical revascularization is increasingly recognized as the mainstay of MMD treatment (Fung et al., 2005). Two large studies with long‐term follow‐up have shown good safety profiles for surgical treatment (Scott et al., 2004; Yamada et al., 1995). A meta‐analysis concluded that 1003 of 1156 patients (87%) received symptomatic benefits from surgical revascularization with indirect, direct, and combined techniques that showed the same effects (Fung et al., 2005). However, the underlying pathogenesis of MMD remains unclear, and current treatment strategies are far from satisfactory. Without prompt diagnosis and effective treatment, numerous patients experience devastating consequences (Kuroda et al., 2005). Therefore, it is critical to develop new methods for the early assessment of cases that are likely to become clinically severe. Early detection of diagnostic biomarkers is essential for improving patient outcomes.

Although the pathogenesis of MMD is unclear, it is increasingly recognized that immunity and inflammation can play an important role in the occurrence and development of MMD (Chen et al., 2015; Huang et al., 2020). In this study, we hypothesized that immunity and inflammation induce characteristic molecular changes that can be detected in the MMD sera. These molecular changes may provide clues for the early diagnosis and treatment of patients with MMD. To test this hypothesis, we used proteomic and metabolomic techniques to analyze the serum proteome and metabolome of patients with MMD and healthy controls (HCs).

## 2 MATERIALS AND METHODS

### 2.1 Patients and samples

Six patients with MMD were selected from the neurology department at the Fifth Medical Center of the Chinese PLA General Hospital, Beijing, China. MMD diagnosis was confirmed by magnetic resonance angiography and imaging, and all patients met the diagnostic criteria of the Research Committee on MMD (spontaneous occlusion of the circle of Willis) of the Ministry of Health, Labor Welfare, Japan, and the Guideline Committee 2021 of the Japan Stroke Society (Fujimura et al., 2022). Patients with other medical conditions, such as central nervous system tumors, severe brain trauma history, previous craniotomy, moyamoya syndrome, hypertension, diabetes, and genetic diseases, were excluded from the study. To establish a control group, the spouses of patients with MMD without a history of neurological disorders were selected. Interested individuals were screened to ensure they met the inclusion criteria, which included being free from any known medical or neurological conditions.

Fasting blood samples were collected from six patients with MMD and six with HC using EDTA‐treated vacuum cleaners. The age of the participants ranged from 18 to 42 years. There were three women and three men in the MMD patient group and three women and three men in the matched HC group (spouses of the patients). Blood collection is performed by coagulation stratification by incubating at 37°C (or room temperature) for 1 h in centrifuge or vacuum collection tubes. The supernatant was transferred to a clean centrifuge tube and centrifuged for 5 min at 3000 rpm. Centrifuge at 12,000 mpm 4°C for 10 min (sampling can also be completed after only one centrifugation), remove the upper clearing and put it in 1.5 mL centrifuge tubes, 0.5 mL per tube, after labeling, liquid nitrogen flash freeze for 15 min, −80°C refrigerator freezing until assay. This study was approved by the Ethics Committee of the Fifth Medical Center of the PLA General Hospital and complied with the Declaration of Helsinki. All patients signed an informed consent form.

### 2.2 Proteomics

#### 2.2.1 Protein extraction and peptide preparation

Protein extraction and peptide preparation were performed according to laboratory protocols. First, each sample was lysed with some DB lysis buffer (8 M urea, 100 mM triethylammonium bicarbonate [TEAB], pH 8.5) and centrifuged at 12,000 × *g* for 20 min at 4°C. This centrifugation step helps to separate the soluble proteins from patient sera. To construct the library for data‐independent acquisition (DIA) protein identification, 20–30 mL of serum from each sample was mixed as a pool sample. The proteins in the pool sample were then separated into high‐ and low‐abundance proteins using the ProteoMinerTM protein enrichment kit (Bio‐Rad). Serum proteins and low‐abundance proteins were then reduced with 2 mM dithiothreitol for 1 h at 56°C and subsequently alkylated with sufficient iodoacetic acid for 1 h at room temperature in the dark. After the precipitation of acetone, the pellets were dissolved using 0.1 M TEAB (pH 8.5) and 8 M urea buffer. The supernatant of each sample containing exactly 0.1 mg of protein was digested with trypsin gold (Promega) at 37°C for 16 h, followed by a desalination procedure to remove high urea, and then dried by vacuum centrifugation.

### 2.3 Library construction

Low‐abundance pool sample peptides were fractionated using a C18 column (Waters BEH C18, 4.6 × 250 mm^2^, 5 m) on a Rigol L‐3000 high‐performance liquid chromatography (HPLC) operating at 1 mL/min. The column oven was set at 50°C. Mobile phases A (2% acetonitrile, adjusted to pH 10.0, using ammonium hydroxide) and B (98% acetonitrile, adjusted to pH 10.0, using ammonium hydroxide) were used for gradient elution. The solvent gradient was set as follows: 3% B, 5 min; 3%–8% B, 0.1 min; 8%–18% B, 11.9 min; 18%–32% B, 11 min; 32%–45% B, 7 min; 45%–80% B, 3 min; 80% B, 5 min; 80%–85%, 0.1 min; and 5% B, 6.9 min. The eluates were monitored at 214 nm, collected in tubes every minute, and merged into four fractions. The four fractions of the low‐ and high‐abundance peptides were dried under vacuum and reconstituted in 0.1% (v/v) formic acid (FA) in water. Then 0.2 L of standard peptides (iRT kit, Biognosys) were added to the peptide samples for subsequent analyses.

For the construction of the transition library, shotgun proteomic analyses were performed using an EASY‐nLC 1200 ultra‐HPLC (UHPLC) system coupled with an Orbitrap Q‐Exactive HF‐X mass spectrometer (Thermo Fisher Scientific) operating in data‐dependent acquisition (DDA) mode. The four fractionated low‐ and high‐abundance peptides reconstituted in 0.1% FA were injected into a homemade C18 Nano‐Trap column (2 cm × 100 µm, 3 µm). The peptides were then separated on a homemade analytical column (15 cm × 150 µm, 1.9 µm), using a 120 min linear gradient from 5% to 100% eluent B (0.1% FA in 80% ACN) in eluent A (0.1% FA in H_2_O) at a flow rate of 600 nL/min. The detailed solvent gradient was as follows: 5%–10% B, 2 min; 10%–40% B, 105 min; 40%–50% B, 5 min; 50%–90% B, 3 min; and 90%–100% B, 5 min.

The Q‐Exactive HF‐X mass spectrometer was operated in positive polarity mode with a spray voltage of 2.3 kV and a capillary temperature of 320°C. Full mass spectrometry (MS) scans ranging from 350 to 1500 *m*/*z* were acquired at a resolution of ×60,000 (at 200 *m*/*z*) with an automatic gain control (AGC) target value of 3 × 10 (Scott et al., 2004) and a maximum ion injection time of 20 ms. The 40 most abundant precursor ions from the full MS scan were selected for fragmentation using higher energy collisional dissociation fragment analysis at a resolution of ×15,000 (at 200 *m*/*z*) with an AGC target value of 5 × 10 (Baba et al., 2008), a maximum ion injection time of 45 ms, a normalized collision energy (NCE) of 27%, an intensity threshold of 2.2 × 10 (Baba et al., 2008), and a dynamic exclusion parameter of 40 s.

### 2.4 MS analysis: DIA mode

Each serum sample peptide was reconstituted in 0.1% FA, mixed with 0.2 µL standard peptides (iRT kit, Biognosys), and injected into the EASY‐nLCTM 1200 UHPLC system coupled with an Orbitrap Q‐Exactive HF‐X mass spectrometer (Thermo Fisher Scientific) operating in DIA mode. The liquid conditions were identical to those used in the DDA model. For DIA acquisition, the MS1 resolution was set to ×60,000 (at 200 *m*/*z*) and the MS2 resolution was set to ×30,000 (at 200 *m*/*z*). The *m*/*z* range was 350–1500 and was separated into 30 acquisition windows (Table S1). The full‐scan AGC target was set to 3 × 10 (Scott et al., 2004) with an injection time of 50 ms. The DIA settings included an NCE of 27%, a target value of 1 × 10 (Scott et al., 2004), and an automatic maximum injection time to allow the MS to operate continuously in parallel ion filling and detection mode.

### 2.5 Proteome data analysis

The raw DIA data was processed and analyzed using Biognosys Spectronaut v.9.0. The software employs MS2‐based label‐free quantification methods to extract quantitative information from the DIA data. This analysis allows for the determination of protein abundance levels across different samples. The analysis process followed the methodology described by Bruder et al. (2015), with minor modifications. After the initial data processing and quantification, further analysis was performed using the Proteome Discoverer (2.2, Thermo Fisher Scientific) platform. This software tool provides a comprehensive range of analytical methods for proteomics data analysis. The R statistical framework was employed for additional data analysis and visualization. To gain insights into the biological pathways and functions associated with the identified proteins, the Kyoto Encyclopedia of Genes and Genomes (KEGG) database was utilized. The enrichment pipeline^16^ was used to perform the KEGG enrichment analysis.

### 2.6 Metabolomics

The samples (100 µL) were placed in EP tubes and resuspended in 80% pre‐chilled methanol by vortexing. The samples were incubated on ice for 5 min and centrifuged at 15,000 × *g*, 4°C for 20 min. Some of the supernatant was diluted to a final concentration containing 53% methanol with LC–MS grade water. The samples were then transferred to a fresh Eppendorf tube and then centrifuged at 15,000 × *g*, 4°C for 20 min. Finally, the supernatant was injected into the LC–MS/MS system analysis. Quality control sample was prepared by mixing equal amounts of the experimental samples. The samples were tested on the machine before, during, and after injection of the LC–MS/MS sample. Samples were loaded using a Hypesil Gold column (100 × 2.1 mm^2^, 1.9 µm) on a Vanquish UHPLC system (Thermo Fisher) coupled with an Orbitrap Q‐Exactive TMHF‐X mass spectrometer (Thermo Fisher). The linear gradient was 17‐min, and the flow rate was 0.2 mL/min. The eluents for the positive polarity mode were eluent A (0.1% FA in water) and eluent B (methanol). The eluents for the negative polarity mode were eluent A (5 mM ammonium acetate, pH 9.0) and eluent B (methanol). The solvent gradient was set as follows: 2% B, 1.5 min; 2%–100% B, 12.0 min; 100% B, 14.0 min; 100‐2% B, 14.1 min; 2% B, 17 min. The raw UHPLC–MS/MS data were analyzed using Compound Discoverer software (version 3.1, Thermo Fisher), R (version 3.4.3), Python (version 2.7.6), and CentOS (version 6.6) to conduct peak alignment, peak picking, and quantitation for each metabolite. Subsequently, the peak intensities were normalized to the total spectral intensity. The peaks were then matched to the mzCloud (https://www.mzcloud.org/), mzVault, and MassList databases to obtain accurate and quantitative results.

### 2.7 Metabolome data analysis

Principal component analysis (PCA) was performed using metaX (a flexible and comprehensive software to process metabolomic data). We applied univariate analysis (*t*‐test) to calculate statistical significance (*p*‐value). Metabolites with VIP > 1 and *p <* .05 and fold change ≥1.5 or FC ≤ 0.5 were considered differential metabolites. Volcano plots were used to filter metabolites of interest based on log2 (fold change) and log10 (*p*‐value) of the metabolites using ggplot2 in R (R Core Team (2022), R: Language and Environment for Statistical Computing, R Foundation for Statistical Computing [https://www.R‐project.org/]). For the clustering heatmaps, the data were normalized using *z*‐scores of the intensity areas of differential metabolites and plotted using the Pheatmap package in R. The correlation between differential metabolites was analyzed using cor() in the R language (method = Pearson). Statistically significant correlations among differential metabolites were calculated using the cor.mtest() in R language. Statistical significance was established at *p <* .05, and correlation plots were plotted using the corrplot package in R, and the enrichment of the metabolic pathway of differential metabolites was performed. For the analysis of the metabolic pathway, metabolites were annotated using the KEGG. When ratios were satisfied by *x*/*n* > *y*/*N*, metabolic pathways were considered enriched, and metabolic pathways were considered statistically significant enrichment.

### 2.8 Statistical analysis

Then all statistical analyses were performed in R (3.5.2). Both metabolomics and proteomics data were converted to Log2 and normalized to median. Use the lmFit and eBay functions implemented in the Limma R package to perform the differential expression (DE) analysis of proteins and metabolites in disease groups (adult MMD and HC, and child MMD and HC). We used multiple comparisons in Limma's design, using all samples from ICM, DCM, and donors to estimate the variance of each protein or metabolite. Then, we compared the two groups of interest to report the direct DE of proteins or metabolites in adult MMD and HC and child MMD and HC. The KEGG annotates pathways (https://www.kegg.jp). Use the pathview online tool (https://Pathview/uncc.edu/overview) to generate a path map. The network diagram of DE protein and metabolites was generated using the igraph R package. The nodes in the network diagram are DE proteins in direct comparison (adult MMD vs. HC and child MMD vs. HC).

## 3 RESULTS

### 3.1 Study design and blood samples

**FIGURE 1:**
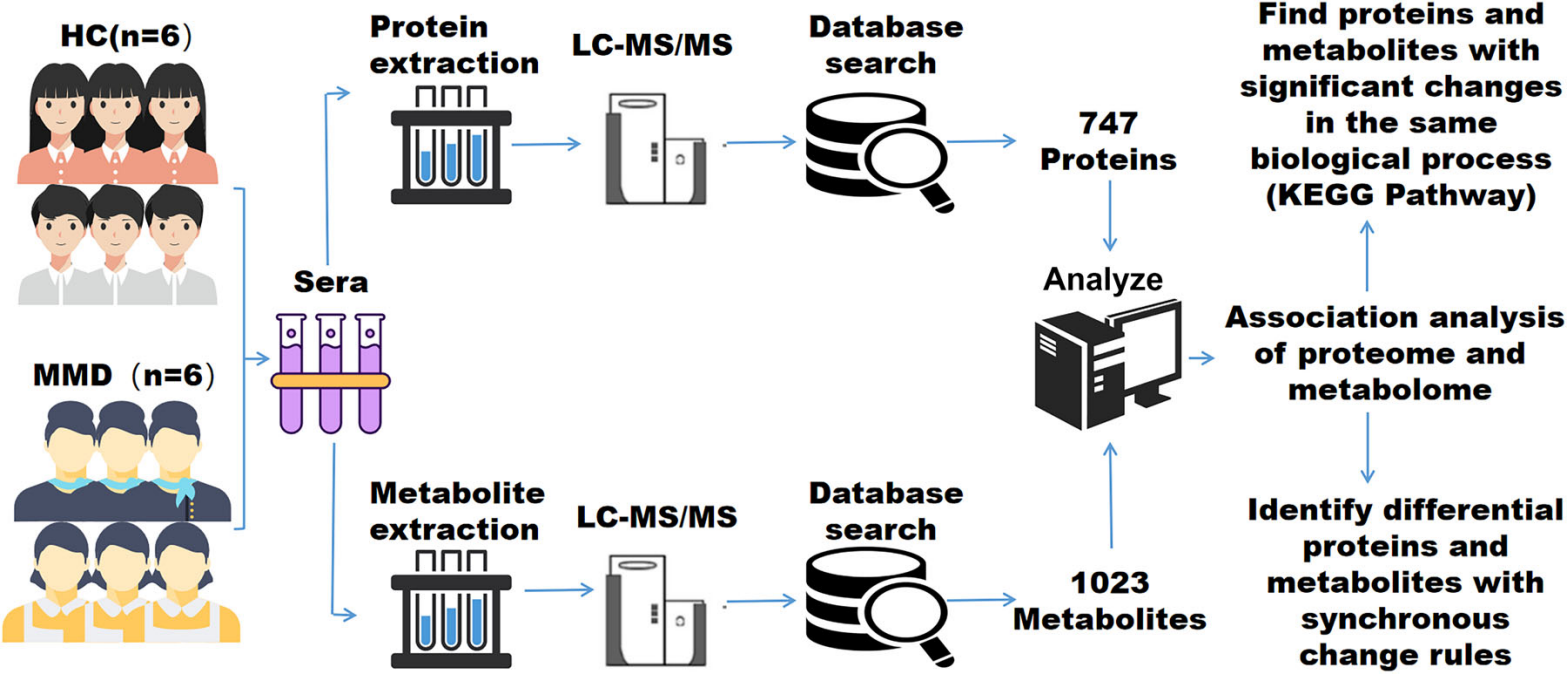
Study design and serum samples. Overview of serum samples collected from adult with moyamoya disease (MMD) (*n* = 6) and adult healthy control (HC) (*n* = 6). It shows the workflow of processing proteomic and metabolomic data, including serum separation, protein extraction, metabolite extraction, LC–MS/MS analysis, database search, and further calculation and analysis.

Blood samples were collected from six adult patients with MMD and six adult HC at the Fifth Medical Center of the PLA General Hospital (Figure 1). All patients with MMD were diagnosed based on the diagnostic criteria of the Research Committee on MMD (Spontaneous Occlusion of Circle of Willis) of the Ministry of Health, Labor Welfare, Japan; the Guideline Committee 2021 of the Japan Stroke Society (Fujimura et al., 2022); and were discharged from the hospital after recovery. The clinical data of the six adult patients with MMD is shown in Table 1, and no severe or critically ill cases were included in our cohort. Six adult HC from the spouses of patients whose habits and eating habits were similar were enrolled for comparison (Table 1).

**TABLE 1:** Clinical baseline of participants.

No/group	Sex	Age	Suzuki stage	Family history	BMI (kg/m^2^)	Diabetes	Hypertension	Medication history
Adult patients with MMD (*n* = 6)								
1	Female	38	V	No	24.6	No	No	No
2	Male	27	V	No	21.7	No	No	No
3	Female	29	IV	No	23.8	No	No	No
4	Male	36	VI	No	23.6	No	No	No
5	Female	41	IV	No	22.7	No	No	No
6	Male	42	VI	No	24.5	No	No	No
Adult healthy controls (*n* = 6)								
1	Male	35			23.9	No	No	No
2	Female	23			21.5	No	No	No
3	Male	32			22.7	No	No	No
4	Female	42			24.7	No	No	No
5	Male	39			25.0	No	No	No
6	Female	35			24.1	No	No	No

Abbreviations: BMI, body mass index; MMD, moyamoya disease.

### 3.2 Proteomics of adult MMD and HC

Quantitative proteomic analysis of serum from patients with MMD and HC was performed using UHPLC–DIA–MS to analyze trypsin peptides, which was combined with a *Homo sapiens* UniProt 2019.01.18. FASTA (169,389 sequences). UHPLC–DIA–MS identified 8967 peptides and quantified 1243.

**FIGURE 2:**
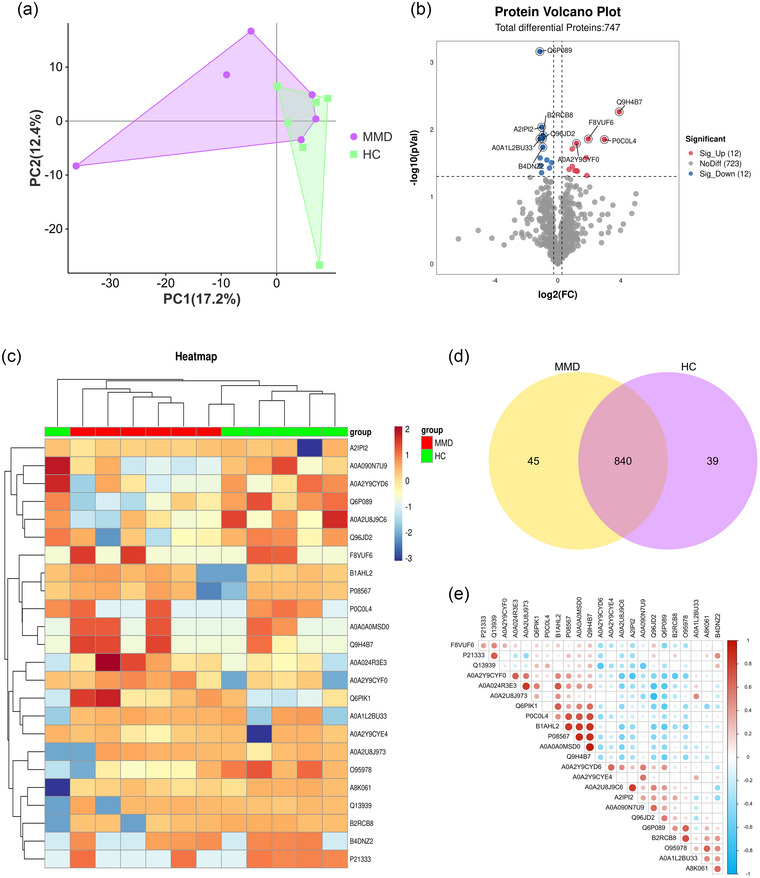
Differential analysis in protein expression levels between moyamoya disease (MMD) and healthy controls (HC): (a) principal component analysis (PCA) of proteomic data in MMD and HC; (b) volcano plot of MMD and HC proteins; (c) multigroup heatmap with dendrogram of differential expression (DE) protein levels incorporating MMD and HC; (d) Venn diagram summarizing the differential and overlapping proteins; (e) correlation heatmap of DE proteins in MMD and HC. *Source*: Data are provided as a source data file.

We preliminarily evaluated the clustering of each group using PCA (Figure 2a). There were 45 and 39 DE proteins between the adult MMD and HC groups, respectively (Figure 2d). Patients with MMD had 24 significantly different proteins compared to HC (*p <* .05) (Figure 2b, Table S1). The heatmap summarizes the differential proteomic data, which are consistent with the PCA data. Hierarchical clustering showed that adult MMD samples were well separated from HC, with some proteins (such as the tubulin beta‐6 [TUBB6] chain) significantly upregulated (Figure 2c). The correlation heatmap showed that adult MMD and HC samples were co‐regulated by a wide range of proteins (Figure 2e).

One of the main DE proteins in adult MMD compared to HC was the TUBB6 chain (human), which increased 14.86‐fold (*p =* .001) (Table 2). The expression of many immune‐related proteins has undergone significant changes (Table 2, Table S1). In total, 24 proteins were significantly different between adult patients with MMD and HC (Figure 2b and Table S1), the top 10 of which are listed in Table 2.

**TABLE 2:** Top 10 differential expression (DE) proteins in moyamoya disease (MMD) versus healthy controls (HC).

Protein id	Protein	FC	log2FC	*p*‐Value	UP/DOWN
Q6P089	IGH@ protein	0.45	−1.14	.001	Down
Q9H4B7	Tubulin beta‐6 chain (human)	14.86	3.89	.005	Up
A2IPI2	HRV Fab N27‐VL	0.49	−1.02	.009	Down
B2RCB8	*Homo sapiens* protocadherin 12	0.52	−0.95	.012	Down
Q96JD2	Amyloid lambda 6 light chain variable region NEG	0.51	−0.97	.014	Down
A0A1L2BU33	Anti‐staphylococcal enterotoxin D heavy chain variable region	0.46	−1.13	.014	Down
F8VUF6	Decorin	3.81	1.93	.014	Up
P0C0L4	Complement C4‐A	7.74	2.95	.014	Up
A0A2Y9CYF0	Ig heavy chain variable region	2.27	1.18	.016	Up
B4DNZ2	cDNA FLJ57132, highly similar Exostosin‐like 2	0.52	−0.95	.018	Down

### 3.3 Metabolomics of adult MMD and HC

**FIGURE 3:**
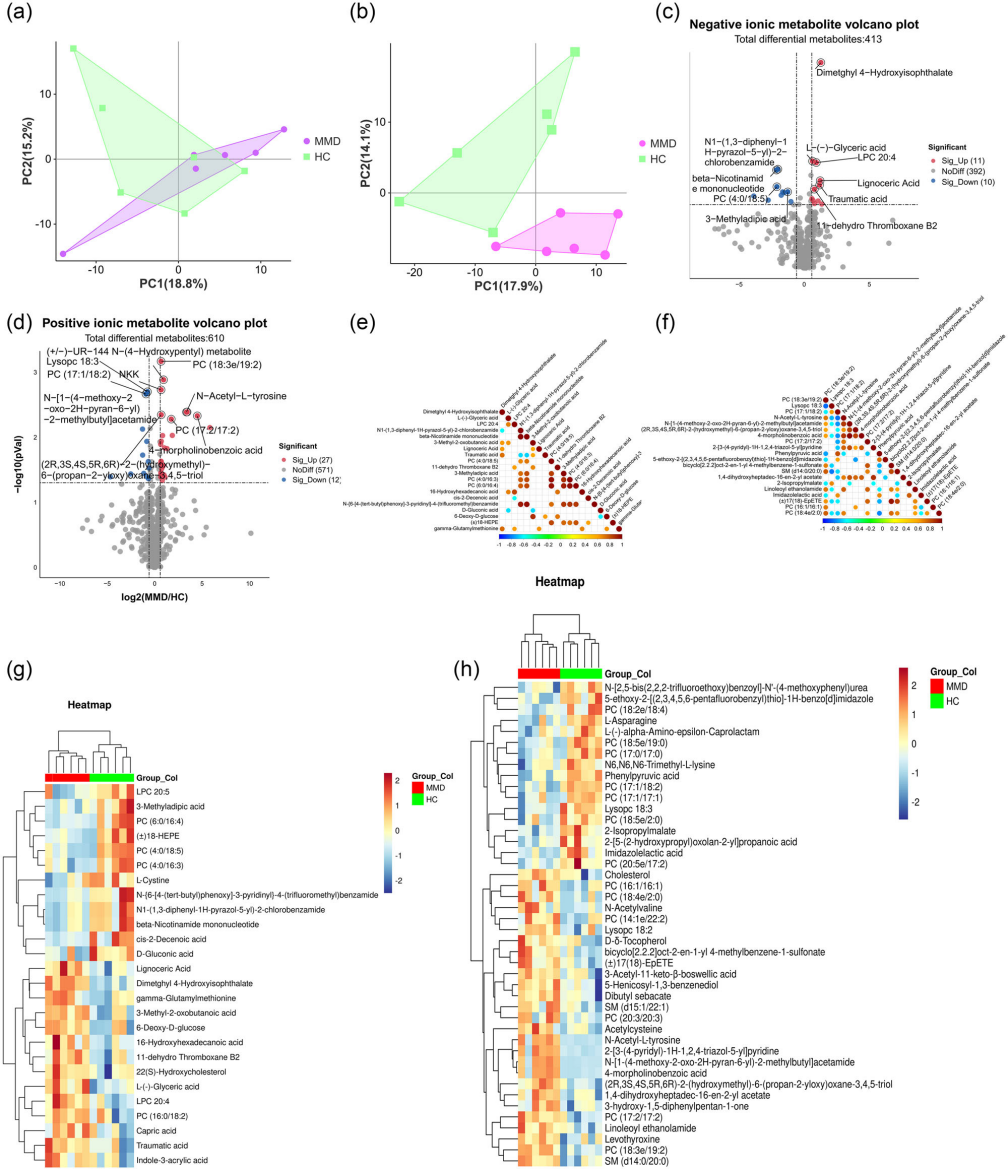
Differential analysis in metabolite abundance levels between moyamoya disease (MMD) and healthy control (HC): (a) principal component analysis (PCA) of negative‐ion metabolomic data for patients with MMD and HC; (b) PCA of positive‐ion metabolomic data from MMD patients and HC; (c) volcano plot of MMD versus HC differential expression (DE) negative‐ion metabolites; (d) volcano plot of MMD versus HC DE positive‐ion metabolites; (e) correlation heatmap of DE negative‐ion metabolites in MMD and HC; (f) correlation heatmap of DE positive‐ion metabolites in the MMD and HC; (g) multigroup heatmap with a dendrogram of DE negative‐ion metabolite levels incorporating MMD and HC; (h) multigroup heatmap with a dendrogram of DE positive‐ion metabolite levels incorporating MMD and HC. *Source*: Data are provided as source data files.

Non‐targeted metabolomic analysis was performed using high‐resolution MS detection technology and identification with an internal library of authentic chemical standards. Like with the proteomic data, we initially evaluated the clustering of the negative‐ and positive‐ion metabolomic data using PCA (Figure 3a,b). The PCA (Figure 3a,b) shows a significant separation between the MMD and HC groups in the overall negative and positive‐ion metabolome. We conducted a two‐group DE analysis to determine differential metabolites regulated between the MMD and HC groups. In total, 21 negative‐ion metabolites and 39 positive‐ion metabolites were DE between the adult MMD and HC groups (*p <* .05, Figure 3c,d and Table S2). The most significant negative ionic DE metabolite in the MMD and HC sera was dimethyl 4‐hydroxyisophthalate (FC = 2.430, *p =* .000) (Table 3). Laboratory and animal studies have shown that dimethyl 4‐hydroxyisophthalate induces oxidative stress (Gourlay et al., 2003; Jepsen et al., 2004) and affects the expression of peroxisome proliferator–activated receptors, suggesting that oxidative stress is a prominent feature of adult MMD. Other negative‐ion metabolites, beta‐nicotinamide mononucleotide (β‐NMN), had the greatest decrease in both MMD and HC (FC = 0.229, *p =* .010) (Table 3). One of the key precursors of nicotinamide adenine dinucleotide (NAD^+^) is β‐NMN, which is the product of the reaction of nicotinamide phosphoribosyl transferase (Nampt). Nampt is an enzyme involved in the mammalian NAD^+^ salvage pathway, which plays an important role in mediating NAD^+^ synthesis in cardiac myocytes. (NAD^+^) has been identified as a key regulator of the life‐extension effect of heat restriction in many species (Imai, 2011). Numerous studies have shown that NAD^+^ mediates a variety of major biological processes in various tissues—like the brain—including calcium homeostasis, mitochondrial function, energy metabolism, aging, and cell death (Xia et al., 2009), suggesting that the reduction of β‐NMN in patients with MMD leads to disorders of multiple metabolic pathways. The most significant positive ionic DE metabolite in the MMD and HC sera was 2‐(3‐(4‐pyridyl)‐1*H*‐1,2,4‐triazol‐5‐yl)pyridine (FC = 56.64, *p =* .007) (Table 4). Another positive‐ion metabolite, PC (17:1/18:2), showed the largest fold change decrease in both the MMD and HC groups (FC = 0.039, *p =* .041) (Table 3). PC (17:1/18:2) is a lecithin that plays an important role in cholesterol metabolism (Canty & Zeisel, 1994). Likewise, PC (18:3e/19:2) (FC = 1.60, *p =* .000), LysoPC 18:3 (FC = 0.59, *p =* .002), *N*‐acetyl‐l‐tyrosine (FC = 10.70, *p =* .004), *N*‐[1‐(4‐methoxy‐2‐oxo‐2*H*‐pyran‐6‐yl)‐2‐methylbutyl]acetamide (FC = 10.20, *p =* .004), (2*R*,3*S*,4*S*,5*R*,6*R*)‐2‐(hydroxymethyl)‐6‐(propan‐2‐yloxy)oxane‐3,4,5‐triol (FC = 1.61, *p =* .005), 4‐morpholinobenzoic acid (FC = 22.59, *p =* .005), and PC (17:2/17:2) (FC = 3.36, *p =* .005) were significantly increased in MMD. Furthermore, phenylpyruvic acid levels decreased in the MMD group (FC = 0.72, *p =* .007) (Table 4).

**TABLE 3:** Top 10 negative‐ion differential expression (DE) metabolites in moyamoya disease (MMD) versus healthy control (HC).

Metabolites	FC	log2FC	*p*‐Value	ROC	Up/Down
Dimethyl 4‐hydroxyisophthalate	2.430	1.281	.000	1.000	Up
l‐(‐)‐Glyceric acid	1.556	0.638	.006	0.972	Up
LPC 20:4	1.910	0.933	.007	0.944	Up
*N*1‐(1,3‐diphenyl‐1*H*‐pyrazol‐5‐yl)‐2‐chlorobenzamide	0.247	−2.017	.009	0.944	Down
beta‐Nicotinamide mononucleotide	0.229	−2.128	.010	0.944	Down
3‐Methyl‐2‐oxobutanoic acid	1.433	0.519	.013	0.944	Up
Lignoceric acid	2.339	1.226	.016	0.944	Up
Traumatic acid	2.271	1.183	.020	0.889	Up
PC (4:0/18:5)	0.234	−2.095	.021	0.833	Down
11‐Dehydro thromboxane B2	1.718	0.781	.024	0.861	Up

**TABLE 4:** Top 10 positive‐ion differential expression (DE) metabolites in moyamoya disease (MMD) versus healthy control (HC).

Metabolites	FC	log2FC	*p*‐Value	ROC	Up/Down
PC (18:3e/19:2)	1.60	0.68	.001	1.000	Down
Lysopc 18:3	0.60	−0.75	.002	1.000	Up
PC (17:1/18:2)	0.53	−0.91	.002	1.000	Up
*N*‐Acetyl‐l‐tyrosine	10.71	3.42	.004	0.944	Up
*N*‐[1‐(4‐methoxy‐2‐oxo‐2*H*‐pyran‐6‐yl)‐2‐methylbutyl] acetamide	10.20	3.35	.004	0.972	Down
(2*R*,3*S*,4*S*,5*R*,6*R*)‐2‐(hydroxymethyl)‐6‐(propan‐2‐yloxy) oxane‐3,4,5‐triol	1.61	0.69	.005	0.944	Down
4‐Morpholinobenzoic acid	22.59	4.50	.005	0.917	Up
PC (17:2/17:2)	3.36	1.75	.005	0.944	Up
2‐[3‐(4‐pyridyl)‐1*H*‐1,2,4‐triazol‐5‐yl]pyridine	56.64	5.82	.007	0.833	Down
Phenylpyruvic acid	0.72	−0.47	.007	0.917	Down

### 3.4 Pathway analyses of DE protein and metabolite

**FIGURE 4:**
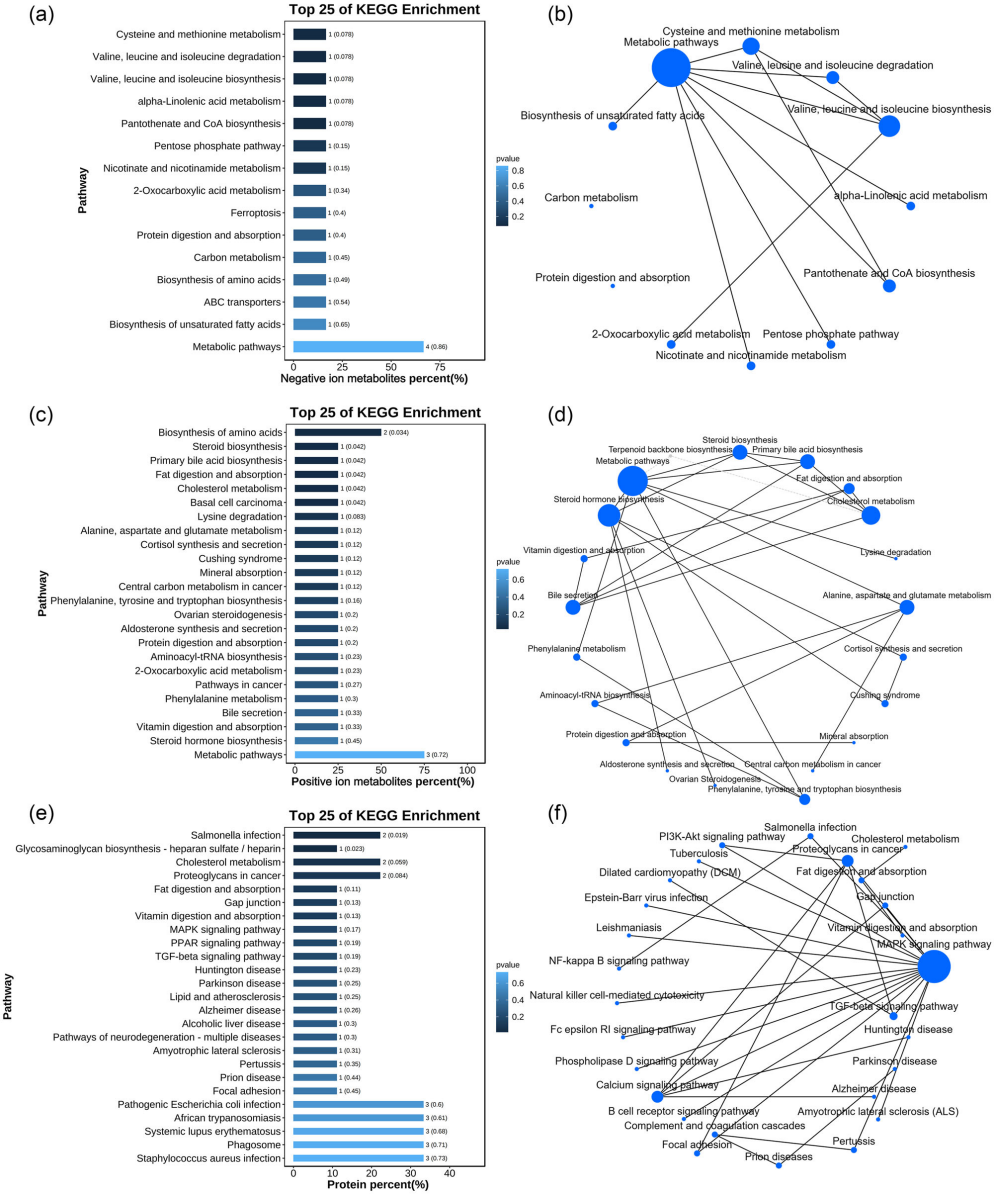
Pathways analysis: (a) Kyoto Encyclopedia of Genes and Genomes (KEGG) enrichment pathways of moyamoya disease (MMD) versus healthy controls (HC) differential expression (DE) negative‐ion metabolites; (b) the network of KEGG pathway annotations of MMD versus HC DE negative‐ion metabolites; (c) KEGG enrichment pathways of MMD versus HC DE positive‐ion metabolites; (d) the network of KEGG pathway annotations of MMD versus HC DE positive‐ion metabolites; (e) KEGG enrichment pathways of MMD versus HC DE proteins; (f) the network of KEGG pathway annotations of MMD versus HC DE proteins. *Source*: Data are provided as a source data file.

Pathway analyses of MMD and HC were performed at differential protein and metabolite levels and annotated using KEGG (Kanehisa & Goto, 2000) (Figure 4). Figure 4a shows the MMD‐regulated pathways at the negative‐ion metabolite level. Fifteen pathways regulate the metabolism of negative ions in MMD. Figure 4b shows the network of KEGG pathway annotations of MMD versus HC DE negative‐ion metabolites. KEGG pathway analysis revealed the important roles of cysteine and methionine metabolism pathways; valine, leucine, and isoleucine biosynthesis pathways; and metabolic pathways in MMD. Figure 4c shows the MMD‐regulated pathways at the positive‐ion metabolite level. There are 24 pathways that regulate the metabolism of positive ions in MMD. Figure 4d shows the network of KEGG pathway annotations for the MMD versus HC DE positive‐ion metabolites. KEGG pathway analysis revealed the important roles of alanine, aspartate, and glutamate metabolism pathways; steroid hormone biosynthesis pathways, metabolic pathways, and cholesterol metabolism pathways in MMD. Figure 4e shows the MMD‐regulated pathways at the protein level. There are 25 pathways that regulate protein metabolism in MMD. Figure 4f shows the network of annotations to the KEGG pathway for the MMD versus HC DE proteins. KEGG pathway analysis revealed the important roles of cholesterol metabolism and proteoglycans in cancer, and the MAPK signaling pathway in MMD.

### 3.5 Integration analysis of DE proteins and metabolites between adult MMD and HC

#### 3.5.1 Correlation analysis of DE protein and metabolite

**FIGURE 5:**
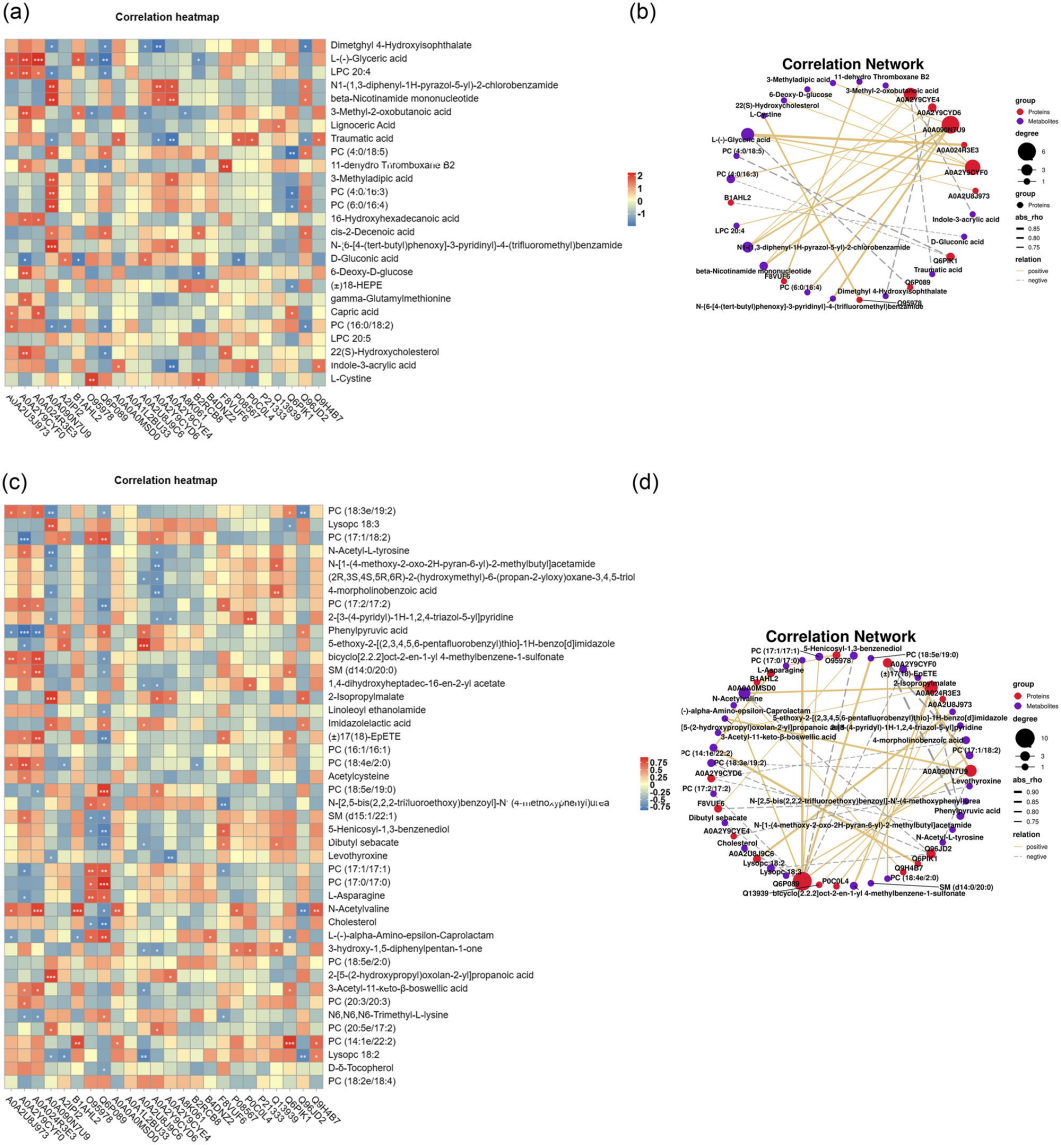
Correlation analyses of differential expression (DE) proteins and DE metabolites: (a) correlation heatmap of DE negative‐ion metabolites and DE proteins in moyamoya disease (MMD) and healthy control (HC); (b) correlation network of DE negative‐ion metabolites and DE proteins in MMD and HC; (c) correlation heatmap of DE positive‐ion metabolites and DE proteins in MMD and HC; (d) correlation network of DE positive‐ion metabolites and DE proteins in MMD and HC. *Source*: Data are provided as a source data file.

Correlation analysis was performed based on Pearson's correlation coefficient among proteins with significant differences in proteomic analysis and negative‐ and positive‐ion metabolites with significant differences in metabolomic analysis to measure the degree of association between differential proteins and differential metabolites (Figure 5c). A0A024R3E3 was strongly positively correlated with l‐(−)‐glyceric acid expression between MMD and HC (correlation coefficient = 0.86, *p =* .000). Q6PIK1 expression was strongly correlated with PC (4:0/18:5) expression in MMD and HC (correlation coefficient = 0.76, *p =* .004). Likewise, A0A090N7U9 and *N*‐{6‐[4‐(*tert*‐butyl)phenox]‐3‐pyridinyl}‐4‐(trifluoromethyl) benzamide, A0A090N7U and 9 β‐NMN, O95978 and l‐cystine, A0A090N7U9s and *N*1‐(1,3‐diphenyl‐1*H*‐pyrazol‐5‐yl)‐2‐chlorobenzamide, F8VUF6 and 11‐dehydro thromboxane B2, A0A2Y9CYF0 and l‐(−)‐glyceric acid, A0A090N7U9 and PC (6:0/16:4), and Q6P089 and l‐(−)‐glyceric acid were strongly correlated (correlation coefficient >0.75, *p <* .05; Table S3). The relationship between metabolites and proteins can be visually represented through a network diagram and metabolites with a screened correlation coefficient greater than 0.7 for mapping (Figure 5b,d).

#### 3.5.2 Integration KEGG pathway analysis of DE proteins and metabolites between adult MMD and HC

**FIGURE 6:**
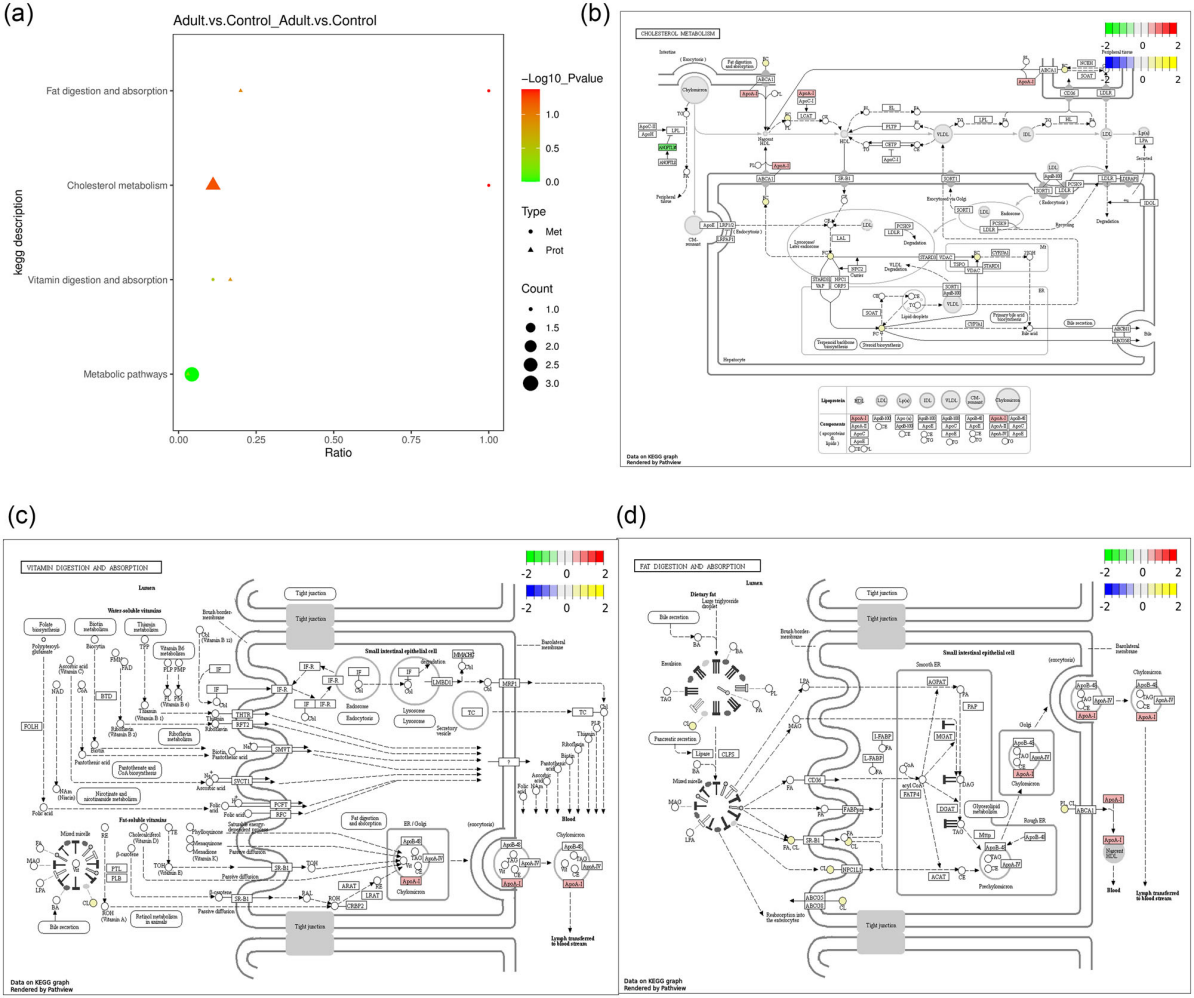
Analysis of share Kyoto Encyclopedia of Genes and Genomes (KEGG) pathway between differential expression (DE) proteins and DE metabolites: (a) share KEGG pathway between DE proteins and DE metabolites; (b) illustrative example of protein and metabolite changes in cholesterol metabolism pathway; (c) illustrative example of protein and metabolite changes in vitamin digestion and absorption pathway; (d) illustrative example of protein and metabolite changes in fat digestion and absorption pathway.

Integration analysis of the KEGG pathway mapped all differential proteins and metabolites to the KEGG pathway database to obtain their common path information and determine the main biochemical and signal transduction pathways involved in the joint participation of differential metabolites and differential proteins (Figure 6a). Figure 6b shows the cholesterol metabolism pathways regulated by MMD at the DE protein and metabolite levels. Figure 6c shows the vitamin digestion and absorption pathways regulated by MMD at the DE protein and metabolite levels. Figure 6d shows the fat digestion and absorption pathways regulated in MMD at the DE protein and metabolite levels.

## 4 DISCUSSION

Besides generating proteomic and metabolomic resources for MMD researchers, we identified possible key disease pathways in MMD compared with age‐, sex‐, and BMI‐matched HC. We identified a well‐established pathogenesis of changes in MMD, including changes in the expression of many inflammatory‐ and immune‐related proteins. This is a recognized feature of MMD pathogenesis in recent years, as well as changes in negative‐ and positive‐ion metabolites. Changes in methionine and cysteine levels suggest that the methionine cycle plays an important role in the pathogenesis of the disease. In MMD, 2‐[3‐(4‐pyridyl)‐1*H*‐1,2,4‐triazol‐5‐yl]pyridine increased 55.64‐fold, which is the first report of this metabolic disturbance in human MMD plasma. It is unclear why the amount of 2‐[3‐(4‐pyridyl)‐1*H*‐1,2,4‐triazol‐5‐yl]pyridine increased significantly. However, it is important to emphasize that it was measured using MMD. Its important derivatives include nicotine and brucine; furthermore, nicotine can accelerate the heart rate, increase blood pressure, damage the intima of blood vessels, and increase the risk of thrombosis (Benowitz & Burbank, 2016).

The underlying pathophysiological mechanisms of MMD remain unknown. From the perspective of vascular injury, angiopathy of MMD is similar to angiopathy of diabetes and arteriosclerosis. Inflammatory pathogenesis plays a crucial role in the vascular cerebral damage observed in various conditions, including diabetes and arteriosclerosis. An increase in inflammatory molecules, like CD40L and MCP‐1, is linked to the later stages of atherosclerotic cerebrovascular disease and raises the likelihood of recurrent cardiovascular events in diabetic patients with stroke (Davì et al., 2009). After 72 h and 7 days of atorvastatin treatment for acute ischemic stroke, the levels of plasma tumor necrosis factor‐α (TNF‐α), interleukin‐6 (IL‐6), and vascular cell adhesion molecule‐1 were significantly decreased, leading to improved prognosis (Tuttolomondo et al., 2016). Interestingly, recent findings indicate that abnormal immune responses to infection are potential triggers for the onset of MMD (Asselman et al., 2022). An increasing number of studies have confirmed that platelet activation occurs in multiple immune‐mediated inflammatory diseases (Asselman et al., 2022; Collins et al., 1994). Platelet activation plays a crucial role in atherosclerosis and atherothrombosis. Noninvasive measurement of plasma thromboxane metabolites can reliably provide biochemical evidence of increased platelet activation in vivo (Simeone et al., 2018). Majumdar et al. (2013) found that stroke with intracranial stenosis is associated with increased platelet activation in patients with sickle cell anemia. In this study, 11‐dehydro Thromboxane B2, a TXT metabolite, increased significantly in MMD (FC = 1.72, *p =* .024). Therefore, we speculate that platelet activation plays a key role in the pathogenesis of immune and inflammatory initiation in MMD. Changes in the Met cycle‐related metabolite levels play a crucial role in the pathogenesis and progression of cardio‐ and cerebrovascular diseases. However, research on the Met cycle‐related metabolites in MMD is scarce. This study showed that l‐cysteine, a Met cycle‐related metabolite, was significantly decreased in MMD (FC = 0.77, *p =* .049). Therefore, Met cycle‐related metabolites may play a crucial role in the pathogenesis and progression of cardiovascular and cerebrovascular diseases. 4‐Morpholinobenzoic acid was significantly increased in MMD (FC = 22.59, *p =* .005). This exacerbates inflammatory reactions by affecting the bile secretion pathways (Wang et al., 2022). Masuda et al. (1993) found vascular smooth muscle cell (VSMC) proliferation, macrophage infiltration, and the presence of T cells in the vascular walls of MMD. Furthermore, the proliferation of ɑ‐adrenoceptor‐dependent VSMCs leads to intimal growth and luminal stenosis, similar in pathological manifestations to those of MMD vessels (Bleeke et al., 2004). In this study, *N*‐acetyl‐l‐tyrosine, an important intermediate product in catecholamine synthesis, was significantly increased in MMD (FC = 10.71, *p =* .004). Thus, catecholamines may play a crucial role in the pathogenesis and progression of MMD.

Our research also provides a basis for the previous observations of the levels of DEP and the discovery of new DEP. TUBB6 is a unique microtubule protein subtype because its overexpression can completely disrupt the microtubule network. Cells with higher TUBB6 expression levels have lower microtubule stability and pyroptosis (Salinas et al., 2014). The leucine‐rich repeat kinase 2 (LRRK2) mutation, which encodes the multifunctional protein LRRK2, is believed to affect the cytoskeleton because the LRRK2 mutant reduces neurite growth and helps to accumulate hyperphosphorylated tau protein. This may lead to changes in the microtubule dynamic instability, which is believed to contribute to the pathogenesis of Parkinson's disease (Law et al., 2014). TUBB6 expression was significantly higher in the MMD group (FC = 14.86, *p =* .005). However, the role of TUBB6 in the pathogenesis of MMD has not yet been elucidated. C4 is a key molecule in the complement system and is one of the main components of innate immunity. It functions through the immediate recognition and elimination of invasive microorganisms. It plays an important role in the classical (CP) and lectin (LP) complement pathways (Wang & Liu, 2021). Furthermore, the overexpression of C4A affected phagocytosis, synaptic pruning, and mouse behavior in microglia. These findings underscore the importance of C4A in synaptic pruning and brain development and highlight the similarities between C4A overexpression and the pathology of schizophrenia (Yilmaz et al., 2021). In this study, C4‐A levels were significantly higher in MMD (FC = 7.74, *p =* .014). Although the exact mechanism of action of C4‐A in the pathogenesis of MMD is unknown, it can be indirectly confirmed that the immune system plays a key role in the pathogenesis of MMD.

The vulnerability of the myocardium to ischemia/reperfusion (I/R) injury is strictly regulated by energy substrate metabolism. Branched‐chain amino acids (BCAA), composed of valine, leucine, and isoleucine, are a group of essential amino acids highly oxidized in the heart. Li et al. (2020) confirmed that chronic accumulation of BCAA enhances glycolysis and fatty acid oxidation (FAO) of ventricular myocytes in adult mice, exacerbates lipid peroxidation toxicity, and increases the vulnerability of the myocardium to I/R injury. Thus, patients with MMD are more prone to cerebral infarctions. In our study, the anion metabolite KEGG was highly enriched, and the valine, leucine, and isoleucine biosynthesis pathways were significantly upregulated.

Disturbances in the alanine, aspartate, and glutamate metabolic pathways can be associated with the development of ischemic stroke by affecting biological processes, such as energy failure, oxidative stress, apoptosis, and glutamate toxicity (Yuan et al., 2022). This follows the results obtained for the MMD. The steroid hormone biosynthesis pathway is significantly upregulated in MMD. Glucocorticoids can also reduce or neutralize the effects of atherosclerosis and vascular injury in chronic inflammatory diseases (Arida et al., 2018). However, glucocorticoids can increase cardiovascular risk by directly and indirectly enhancing metabolic syndrome and vascular damage (Gami et al., 2007). Proteoglycans are key molecular effectors on the cell surface and the extracellular microenvironment. Due to their polyhedral nature and ability to interact with ligands and receptors that regulate tumor growth and angiogenesis, they play a variety of functions in cancer and angiogenesis. Furthermore, some proteoglycans, such as polysaccharides, have pro‐ and antiangiogenic functions. Proteoglycans in the cancer pathway are significantly upregulated in MMD, and the upregulation of the MAPK signaling pathway promotes the response of aging (in vitro) and aging (in vivo, animal models, and human cohorts) to oxidative stress and inflammation. These networks promote age‐related cardiovascular diseases (Papaconstantinou, 2019). In this study, the MAPK signaling pathway is significantly upregulated in MMD.

Biological phenomena and the regulation of gene expression are complex and variable. When conducting single‐omics research, the conclusions are often not sufficiently comprehensive; therefore, there are bottlenecks. Multiomics technology combines two or more histological research methods, such as proteomics or metabolomics, to conduct systematic research on biological samples. Multiomics integration analysis normalizes batch data from different biomolecular levels to establish relationships among molecules at different levels. Combined with biological function analysis, such as KEGG pathway enrichment and molecular interactions, we systematically and comprehensively analyzed biological molecular functions and regulatory mechanisms. To the best of our knowledge, no integrated analysis of the MMD has yet been reported. Although metabolomics and proteomics may have relatively unique profiles, we found that the DE proteins and metabolites were simultaneously associated with cholesterol metabolism, vitamin digestion and absorption, and fat digestion and absorption pathways in MMD.

Cholesterol metabolism is a complex and interesting process. It is well known that it is closely related to unhealthy diet and arteriosclerosis. However, recent studies have confirmed that cholesterol metabolism affects the immune response and the ability of organisms to clear infections and tumor cells while maintaining balance and health in the body (Cardoso & Perucha, 2021). The accumulation of cholesterol supports the inflammatory response of myeloid cells, which is beneficial to the response to infection, but worsens diseases related to chronic metabolic inflammation, including atherosclerosis. In addition to the innate immune system, cells with adaptive immune functions have been shown to undergo reprogramming of cellular cholesterol metabolism after activation, leading to the amplification of inflammatory reactions (Yvan‐Charvet et al., 2019). Platelet activation increases the risk 13 of atherosclerosis and thrombosis by affecting atherosclerotic lesion cells such as macrophages. Increasing evidence indicates that hypercholesterolemia increases the risk of atherosclerosis and thrombosis by regulating platelet biogenesis and activity of platelets (Wang & Tall, 2016). In this study, DE proteins and metabolites were simultaneously enriched in the cholesterol metabolism pathway. Therefore, we speculate that the cholesterol metabolic pathway plays a key role in immune metabolic disorders in MMD. Increased homocysteine (Hcy) levels can lead to other types of vascular damage. Hcy is an intermediate metabolite of 1‐carbon metabolism that promotes endothelial dysfunction, oxidative stress, inflammation, cell proliferation, and thrombosis (McCully, 2009). Hcy metabolism depends on nutritional factors that include folic acid, vitamin B12, choline, riboflavin, and vitamin B6 (Clarke, 2000; Selhub et al., 1993). Most observational studies have concluded that moderate Hcy levels are associated with CVD risk. These studies indicate that a moderate increase in the Hcy level is a risk factor for CV mortality, ischemic heart disease, peripheral arterial disease, and venous thrombosis (Alfthan et al., 1997; Welch & Loscalzo, 1998). Hyperhomocysteinemia, caused by a lack of folic acid and methionine in a monkey diet, induces endothelial dysfunction, which is characterized by an impaired vasodilation response to acetylcholine or adenosine diphosphate, decreased anticoagulant activity of thrombomodulin, and increased platelet‐mediated collagen infusion vasoconstriction (Lentz et al., 1996). Vitamin digestion and absorption play a key role in these processes. These processes are like those observed in the pathogenesis of MMD. Therefore, vitamin digestion and absorption may play a key role in the pathogenesis of MMD. In individuals with low‐to‐moderate cardiovascular risk, subclinical atherosclerosis and vascular inflammation are associated with hypertriglyceridemia, even among those with normal levels of low density lipoprotein (LDL) cholesterol (Raposeiras‐Roubin et al., 2021). Immune cell activation and the subsequent initiation of an inflammatory cascade are initiated by the infiltration of apolipoproteins into the endo subcutaneous space. LDL, through oxidative or enzymatic modification of the inner membrane, triggers the release of oxidized phospholipids, which have been shown to activate endothelial cells (Leitinger, 2003), leading to the increased expression of adhesion molecules and inflammatory genes. Reactive oxygen species (such as superoxide anion, peroxide radical, and peroxynitrite) upregulate NF‐κB expression, a multifunctional transcription regulator of inflammatory gene expression, and reduce the biological activity of nitric oxide, an inhibitor of NF‐κB (Deanfield et al., 2005). Pollaci et al. determined the biochemical and functional characteristics of RNF213 R4180K mutations in angiogenesis through in vitro and in vivo studies in 2015. They found that the up‐regulation of RNF213 can be caused by inflammatory signals (Pollaci et al., 2022). The inflammatory pathways activated by fat metabolism may play an important role in the initiation and progression of MMD.

Although this is the first MMD study to integrate proteomics and metabolomics, it has several limitations. First, proteomic and metabolomic data may require a larger sample size, as our sample size was considerably small. Second, the selected patients were from a Chinese center with obvious regional characteristics. Third, to ensure consistency in the sample, our age matching was for young adult patients; therefore, it cannot fully represent the entire population with MMD. Fourth, various factors, such as diet and environment, may affect plasma metabolites. MMD was associated with cholesterol, fat, and vitamin metabolism. These metabolic pathways are like the metabolism of arteriosclerosis; however, the pathological mechanism of MMD differs from that of arteriosclerosis. Finally, these results must be validated in a larger independent patient cohort; metabolites must be confirmed with their chemical standards, and all findings should be further confirmed using experimental models.

## 5 CONCLUSIONS

Proteomics and untargeted metabolomics studies of plasma samples from patients with MMD and HC identified 24 proteins and 50 metabolites that differed significantly during the progression of MMD, including 21 anionic metabolites and 39 cationic metabolites. Through pathway enrichment analysis, we found that cysteine and methionine metabolism pathways; valine, leucine, and isoleucine biosynthesis pathways; cholesterol metabolism; proteoglycans in cancer; and MAPK signaling pathways are all closely related to MMD. Further integrated proteomics and metabolomics studies were conducted to determine cholesterol metabolism, vitamin digestion, and absorption pathways; and fat digestion and absorption pathways are the share pathway of proteins and metabolites and are worthy of further study.

## AUTHOR CONTRIBUTIONS

Qingbao Guo, Qian‐Nan Wang, and Jingjie Li performed the experimental research and data analysis and wrote and edited the manuscript. Simeng Liu, Xiaopeng Wang, Yu, Hao, Gao, Zhang, Zheng‐Xing Zou, and Jie Feng collected the samples. Ri‐Miao Yang, Minjie Wang, Heguan Fu, Xiangyang Bao, and Lian Duan contributed to the study design, data analysis, and manuscript writing and editing. All authors have read and approved the final manuscript, have full access to all data in the study, and take responsibility for the integrity and security of the data.

## CONFLICT OF INTEREST STATEMENT

The authors declare no conflicts of interest.

### PEER REVIEW

The peer review history for this article is available at https://publons.com/publon/10.1002/brb3.3328

## Supporting information

Supplementary table 1 24 DE proteins in MMD vs HC
Supplementary table 2 21 negative ion DE metabolites in MMD vs HC
Supplementary table 3 39 positive ion DE metabolites in MMD vs HC

## Data Availability

Our team offers proteomic results via a user‐friendly online repository (https://www.iprox.org/) as a publicly available resource, thereby enabling researchers without access to MMD patient sera. Metabolomics results were generated and stored at the Fifth Medical Center of the People's Liberation Army of China (PLA) General Hospital. Data supporting the results of this study may be obtained from the corresponding authors if the requirements are reasonable.
