# Supplementary material for: Proteomic and metabolomic characterizations of moyamoya disease patient sera
Source: Brain Behav. 2023 Nov 14;13(12):e3328. doi: 10.1002/brb3.3328 (PMC10726768; doi:10.1002/brb3.3328)
Supplement: Supplementary file 1 — Supplementary table 1 24 DE proteins in MMD vs HC Supplementary table 2 21 negative ion DE metabolites in MMD vs HC Supplementary table 3 39 positive ion DE metabolites in MMD vs HC [file BRB3-13-e3328-s001.docx]

| **Supplementary table 1 24 DE proteins in MMD vs HC** | | | | | |
| --- | --- | --- | --- | --- | --- |
| **Protein id** | **Protein** | **FC** | **log2FC** | **Pvalue** | **UP.DOWN** |
| A0A090N7U9 | Retinoic acid receptor responder (Tazarotene induced) 2 | 0.76 | -0.39 | 0.031 | down |
| A0A0A0MSD0 | Sushi, von Willebrand factor type A, EGF and pentraxin domain-containing protein 1 | 3.49 | 1.80 | 0.026 | up |
| A0A1L2BU33 | Anti-staphylococcal enterotoxin D heavy chain variable region (Fragment) | 0.46 | -1.13 | 0.014 | down |
| A0A2U8J973 | Ig heavy chain variable region (Fragment) | 2.27 | 1.18 | 0.041 | up |
| A0A2U8J9C6 | Ig heavy chain variable region (Fragment) | 0.69 | -0.53 | 0.037 | down |
| A0A2Y9CYD6 | Ig heavy chain variable region (Fragment) | 0.62 | -0.70 | 0.028 | down |
| A0A2Y9CYE4 | Ig heavy chain variable region (Fragment) | 0.49 | -1.03 | 0.044 | down |
| A0A2Y9CYF0 | Ig heavy chain variable region (Fragment) | 2.27 | 1.18 | 0.016 | up |
| A2IPI2 | HRV Fab N27-VL (Fragment) | 0.49 | -1.02 | 0.009 | down |
| A8K061 | cDNA FLJ77880, highly similar to Homo sapiens angiopoietin-like 3, mRNA | 0.46 | -1.13 | 0.027 | down |
| B1AHL2 | Fibulin-1 | 3.59 | 1.85 | 0.048 | up |
| B2RCB8 | cDNA, FLJ95971, highly similar to Homo sapiens protocadherin 12 (PCDH12), mRNA | 0.52 | -0.95 | 0.012 | down |
| B4DNZ2 | cDNA FLJ57132, highly similar to Exostosin-like 2 | 0.52 | -0.95 | 0.018 | down |
| F8VUF6 | Decorin (Fragment) | 3.81 | 1.93 | 0.014 | up |
| O95978 | VH1 protein (Fragment) | 0.47 | -1.10 | 0.034 | down |
| P08567 | Pleckstrin | 2.36 | 1.24 | 0.042 | up |
| P0C0L4 | Complement C4-A | 7.74 | 2.95 | 0.014 | up |
| P21333 | Filamin-A | 2.12 | 1.08 | 0.041 | up |
| Q13939 | Calicin | 1.90 | 0.92 | 0.035 | up |
| Q6P089 | IGH@ protein | 0.45 | -1.14 | 0.001 | down |
| Q6PIK1 | IGL@ protein | 1.88 | 0.91 | 0.020 | up |
| Q96JD2 | Amyloid lambda 6 light chain variable region NEG (Fragment) | 0.51 | -0.97 | 0.014 | down |
| Q9H4B7 | Tubulin beta-1 chain | 14.86 | 3.89 | 0.005 | up |

| **Supplementary table 2 21 negative ion DE metabolites in MMD vs HC** | | | | | |
| --- | --- | --- | --- | --- | --- |
| **Metabolites** | **FC** | **log2FC** | **Pvalue** | **ROC** | **Up.Down** |
| Dimetghyl 4-Hydroxyisophthalate | 2.43 | 1.28 | 6.2E-05 | 1 | up |
| L-(-)-Glyceric acid | 1.56 | 0.64 | 6.4E-03 | 0.972 | up |
| LPC 20:4 | 1.91 | 0.93 | 6.9E-03 | 0.944 | up |
| N1-(1,3-diphenyl-1H-pyrazol-5-yl)-2-chlorobenzamide | 0.25 | -2.02 | 9.4E-03 | 0.944 | down |
| beta-Nicotinamide mononucleotide | 0.23 | -2.13 | 9.8E-03 | 0.944 | down |
| 3-Methyl-2-oxobutanoic acid | 1.43 | 0.52 | 1.3E-02 | 0.944 | up |
| Lignoceric Acid | 2.34 | 1.23 | 1.6E-02 | 0.944 | up |
| Traumatic acid | 2.27 | 1.18 | 2.0E-02 | 0.889 | up |
| PC (4:0/18:5) | 0.23 | -2.09 | 2.1E-02 | 0.833 | down |
| 11-dehydro Thromboxane B2 | 1.72 | 0.78 | 2.4E-02 | 0.861 | up |
| 3-Methyladipic acid | 0.42 | -1.27 | 2.7E-02 | 0.889 | down |
| PC (4:0/16:3) | 0.32 | -1.65 | 2.7E-02 | 0.833 | down |
| PC (6:0/16:4) | 0.34 | -1.55 | 2.8E-02 | 0.861 | down |
| 16-Hydroxyhexadecanoic acid | 1.32 | 0.40 | 3.0E-02 | 0.917 | up |
| cis-2-Decenoic acid | 0.29 | -1.76 | 3.2E-02 | 0.833 | down |
| N-{6-[4-(tert-butyl)phenoxy]-3-pyridinyl}-4-(trifluoromethyl)benzamide | 0.07 | -3.87 | 3.3E-02 | 0.944 | down |
| D-Gluconic acid | 0.81 | -0.31 | 3.6E-02 | 0.889 | down |
| 6-Deoxy-D-glucose | 1.51 | 0.60 | 3.8E-02 | 0.833 | up |
| (±)18-HEPE | 0.15 | -2.76 | 4.0E-02 | 0.833 | down |
| gamma-Glutamylmethionine | 1.60 | 0.68 | 4.1E-02 | 0.833 | up |
| Capric acid | 2.10 | 1.07 | 4.2E-02 | 0.861 | up |
| PC (16:0/18:2) | 1.42 | 0.51 | 4.2E-02 | 0.833 | up |
| LPC 20:5 | 0.49 | -1.02 | 4.3E-02 | 0.889 | down |
| 22(S)-Hydroxycholesterol | 1.73 | 0.79 | 4.7E-02 | 0.972 | up |
| Indole-3-acrylic acid | 2.55 | 1.35 | 4.9E-02 | 0.861 | up |
| L-Cystine | 0.76 | -0.39 | 4.9E-02 | 0.861 | down |

| **Supplementary table 3 39 positive ion DE metabolites in MMD vs HC** | | | | | | |
| --- | --- | --- | --- | --- | --- | --- |
| **Metabolites** | **FC** | **log2FC** | | **Pvalue** | **ROC** | **Up.Down** |
| PC (18:3e/19:2) | 1.60 | 0.68 | 0.001 | | 1.000 | up |
| Lysopc 18:3 | 0.59 | -0.75 | | 0.002 | 1.000 | down |
| PC (17:1/18:2) | 0.53 | -0.91 | | 0.002 | 1.000 | down |
| N-Acetyl-L-tyrosine | 10.71 | 3.42 | | 0.004 | 0.944 | up |
| N-[1-(4-methoxy-2-oxo-2H-pyran-6-yl)-2-methylbutyl]acetamide | 10.20 | 3.35 | | 0.004 | 0.972 | up |
| (2R,3S,4S,5R,6R)-2-(hydroxymethyl)-6-(propan-2-yloxy)oxane-3,4,5-triol | 1.61 | 0.69 | | 0.005 | 0.944 | up |
| 4-morpholinobenzoic acid | 22.59 | 4.50 | | 0.005 | 0.917 | up |
| PC (17:2/17:2) | 3.36 | 1.75 | | 0.005 | 0.944 | up |
| 2-[3-(4-pyridyl)-1H-1,2,4-triazol-5-yl]pyridine | 56.64 | 5.82 | | 0.007 | 0.833 | up |
| Phenylpyruvic acid | 0.72 | -0.47 | | 0.007 | 0.917 | down |
| 5-ethoxy-2-[(2,3,4,5,6-pentafluorobenzyl)thio]-1H-benzo[d]imidazole | 0.41 | -1.28 | | 0.007 | 0.944 | down |
| bicyclo[2.2.2]oct-2-en-1-yl 4-methylbenzene-1-sulfonate | 3.63 | 1.86 | | 0.009 | 0.944 | up |
| SM (d14:0/20:0) | 1.78 | 0.83 | | 0.009 | 0.944 | up |
| 1,4-dihydroxyheptadec-16-en-2-yl acetate | 1.62 | 0.70 | | 0.012 | 0.917 | up |
| 2-Isopropylmalate | 0.56 | -0.83 | | 0.012 | 1.000 | down |
| Linoleoyl ethanolamide | 1.56 | 0.64 | | 0.013 | 0.917 | up |
| Imidazolelactic acid | 0.73 | -0.45 | | 0.014 | 0.917 | down |
| (±)17(18)-EpETE | 2.04 | 1.03 | | 0.015 | 0.917 | up |
| PC (16:1/16:1) | 2.46 | 1.30 | | 0.015 | 0.917 | up |
| PC (18:4e/2:0) | 1.45 | 0.54 | | 0.018 | 0.889 | up |
| Acetylcysteine | 1.61 | 0.68 | | 0.024 | 0.917 | up |
| PC (18:5e/19:0) | 0.63 | -0.67 | | 0.024 | 0.889 | down |
| N-[2,5-bis(2,2,2-trifluoroethoxy)benzoyl]-N'-(4-methoxyphenyl)urea | 0.80 | -0.32 | | 0.024 | 0.861 | down |
| SM (d15:1/22:1) | 1.56 | 0.64 | | 0.025 | 0.861 | up |
| 5-Henicosyl-1,3-benzenediol | 1.80 | 0.85 | | 0.027 | 0.944 | up |
| Dibutyl sebacate | 1.82 | 0.87 | | 0.028 | 0.972 | up |
| Levothyroxine | 1.34 | 0.42 | | 0.028 | 0.833 | up |
| PC (17:1/17:1) | 0.77 | -0.37 | | 0.029 | 0.861 | down |
| PC (17:0/17:0) | 0.67 | -0.59 | | 0.030 | 0.861 | down |
| L-Asparagine | 0.68 | -0.55 | | 0.030 | 0.861 | down |
| N-Acetylvaline | 1.67 | 0.74 | | 0.031 | 0.861 | up |
| Cholesterol | 1.38 | 0.46 | | 0.031 | 1.000 | up |
| L-(-)-alpha-Amino-epsilon-Caprolactam | 0.73 | -0.45 | | 0.034 | 0.861 | down |
| 3-hydroxy-1,5-diphenylpentan-1-one | 1.57 | 0.65 | | 0.035 | 0.833 | up |
| PC (18:5e/2:0) | 0.45 | -1.15 | | 0.036 | 0.889 | down |
| 2-[5-(2-hydroxypropyl)oxolan-2-yl]propanoic acid | 0.17 | -2.52 | | 0.037 | 0.944 | down |
| 3-Acetyl-11-keto-β-boswellic acid | 1.58 | 0.66 | | 0.037 | 0.889 | up |
| PC (20:3/20:3) | 1.99 | 0.99 | | 0.038 | 0.806 | up |
| N6,N6,N6-Trimethyl-L-lysine | 0.69 | -0.53 | | 0.041 | 0.917 | down |
| PC (20:5e/17:2) | 0.04 | -4.68 | | 0.041 | 0.917 | down |
| PC (14:1e/22:2) | 2.13 | 1.09 | | 0.041 | 0.806 | up |
| Lysopc 18:2 | 1.27 | 0.35 | | 0.042 | 0.833 | up |
| D-δ-Tocopherol | 1.75 | 0.80 | | 0.046 | 0.833 | up |
| PC (18:2e/18:4) | 0.73 | -0.45 | | 0.046 | 0.806 | down |
